# A winning formula: sustainable control of three stored-product insects through paired combinations of entomopathogenic fungus, diatomaceous earth, and lambda-cyhalothrin

**DOI:** 10.1007/s11356-024-31824-1

**Published:** 2024-01-31

**Authors:** Waqas Wakil, Nickolas G. Kavallieratos, Nikoleta Eleftheriadou, Syed Adnan Haider, Mirza Abdul Qayyum, Muhammad Tahir, Khawaja G. Rasool, Mureed Husain, Abdulrahman S. Aldawood

**Affiliations:** 1 Department of Entomology, University of Agriculture, Faisalabad, 38040 Pakistan; 2 Senckenberg German Entomological Institute, 15374 Müncheberg, Germany; 3 Laboratory of Agricultural Zoology and Entomology, Department of Crop Science, Agricultural University of Athens, 75 Iera Odos str, 11855 Athens, Greece; 4 Institute of Plant Protection, Muhammad Nawaz Shareef University of Agriculture, Multan, Pakistan; 5 National Agricultural Research Centre, Ministry of National Food Security and Research, Islamabad, 44000 Pakistan; 6 Department of Plant Protection, College of Food and Agriculture Sciences, King Saud University, P.O. Box 2460, 11451 Riyadh, Saudi Arabia

**Keywords:** Insecticide combinations, Pyrethroid, Entomopathogenic fungus, Diatomaceous earth, Stored grain, Pest management

## Abstract

This research aimed to assess the effectiveness of *Metarhizium robertsii*, diatomaceous earth (Protect-It), and lambda-cyhalothrin, for the long-term protection of stored wheat against three destructive grain insect pests, *Rhyzopertha dominica*, *Tribolium castaneum*, and *Trogoderma granarium*. Different treatments were applied, both alone and in paired combinations in laboratory and persistence trials. Single treatments exhibited significantly lower mortality rates in comparison to the paired treatments for all tested insect species. Among the single treatments, lambda-cyhalothrin (Lamb) resulted in significantly higher mortality rates in laboratory trials, followed by diatomaceous earth (DE) and *M. robertsii* (Mr), with insignificant differences between Mr and DE. Evidently, DE exhibited the highest persistence after 120 days of storage for all insect species and initial exposures, although variations in mortality rates among treatments were mostly insignificant. Overall, the most effective treatment in terms of mortality in laboratory, and persistence trials, and progeny production was DE + Lamb, followed by Mr + Lamb, and Mr + DE for all tested insect species. In general, the most susceptible insect species was *R. dominica*, followed by *T. castaneum* and *T. granarium*. This research highlights the effectiveness of *M. robertsii*, DE, and lambda-cyhalothrin in providing prolonged protection of stored wheat against all the examined grain insect species.

## Introduction

In the pursuit of sustenance and shelter, various insect pests pose a significant threat to stored grains, causing both qualitative and quantitative losses (Padin et al. 2002). Pests can infiltrate grain storage facilities during various stages of grain processing, yard threshing, and transit (Deshwal et al. 2020). Their spread is facilitated by grain movement, both passively and through the active flight of certain adult insects. Globally, over 1600 insect species have been associated with stored products (Hagstrum et al. 2013). These pests damage grains and contaminate them, adversely affecting their taste and smell, and rendering them unsuitable for consumption (Rajashekar et al. 2012; Hagstrum et al. 2013; Said and Pashte 2015). *Rhyzopertha dominica* (F.) (Coleoptera: Bostrychidae), *Tribolium castaneum* (Herbst) (Coleoptera: Tenebrionidae), and *Trogoderma granarium* Everts (Coleoptera: Dermestidae) constitute some of the most common insect pests of stored products. Wheat, cereals, and various other commodities worldwide are subjected to deterioration caused by these voracious pests, resulting in significant grain losses and a decline in product quality (Rees 2004; Hagstrum et al. 2013; Đukić et al. 2016, 2020). *Rhyzopertha dominica* is recognized as a primary insect pest that affects various commodities globally, including cereals, corn, rice, and wheat. This pest directly consumes the germ and endosperm of grains, and its adult stage can fly between agricultural and non-agricultural areas (Mahroof et al. 2010; Hagstrum et al. 2013; Majeed et al. 2015). *Tribolium castaneum* is considered a secondary pest with a global distribution, infesting a wide range of up to 233 different stored products (Hagstrum et al. 2013; Suleiman and Rosentrater 2022). *Trogoderma granarium* holds the status of a primary pest and is listed among the 100 most invasive species worldwide, while this pest falls under the A1 quarantine category of the EPPO (Lowe et al. 2000; EPPO 2023). The urgent need for pest control has driven a widespread reliance on chemical pesticides, notably organophosphates and pyrethroids, for grain protection. However, this relentless dependence has led to a concerning development in contemporary agriculture: the emergence of resistance among various storage pests. The well-established obstacle of resistance has significantly eroded the effectiveness of conventional pest management practices (Attia et al. 2020; Yadav et al. 2020; Cui et al. 2021; Amjad et al. 2022). The constraints associated with pesticide use have prompted scientists to explore alternative control measures, encompassing botanicals, inert dusts, insect growth regulators, and microbial agents as integral components of integrated pest management (IPM) strategies (Shah and Khan 2014).

Diatomaceous earths (DEs) present a viable alternative to conventional contact insecticides. DEs consist of fossilized diatoms, phytoplankton organisms with a long history dating back to the Eocene and Miocene eras, forming intricate silica-based skeletons. After their demise, these silica shells accumulate on the ocean floor, creating extensive layers of fossilized diatom shells, resulting in a soft sedimentary rock known as DE (Martinovic et al. 2006; Hadjar et al. 2008; Sağlam et al. 2017; Losic and Korunic 2018). DEs are characterized by their unique physical mode of action against insect pests. They desiccate insects by disrupting their waterproof outer layer, inducing water loss and eventual pest mortality (Subramanyam and Roesli 2000; Losic and Korunic 2018; Korunić et al. 2020). The toxicity of DEs primarily arises from their physical attributes rather than chemical composition. Mechanisms underlying their insecticidal efficacy include the absorption of cuticular waxes, cuticle abrasion, damage to the digestive tract, surface enlargement through dehydration, and the obstruction of spiracles and tracheae (Subramanyam and Roesli 2000; Losic and Korunic 2018). DEs demonstrate minimal toxicity to vertebrates (Vurro et al. 2019; Audu and Ibrahim 2021), exhibit high efficacy against insects (Korunić et al. 2016; Losic and Korunic 2018), and leave no residues on food products since they can be easily removed from grain surfaces before the milling process (Subramanyam and Roesli 2000; Losic and Korunic 2018).

In the context of limiting pesticide use, the incorporation of biopesticides, such as entomopathogenic fungi (EPF), within the framework of IPM programs can offer significant advantages. EPF, which comprise over 750 species (Rajula et al. 2020), have demonstrated their potential as biocontrol agents against various insect pests found in storage facilities (Batta and Kavallieratos 2018; Ak 2019). Some EPF species are considered suitable candidates for the formulation of mycoinsecticides due to their high virulence and low toxicity to mammals (Shah and Pell 2003; Batta and Kavallieratos 2018; Wakil et al. 2010; Gad et al. 2020). A notable advantage of EPF is their extended residual persistence compared to conventional insecticides (Moore et al. 2012). The infection process initiated by EPF involves spore attachment to the target insect, followed by spore penetration through the insect's cuticle via the secretion of specific cuticle-degrading enzymes. Subsequently, the insect's body is colonized by hyphae, which release mycotoxins, ultimately leading to the demise of the targeted insect pest within a few days (Trinh et al. 2020; Wang et al. 2021). EPF belonging to the genera *Lecanicillium* (Hypocreales: Cordycipitaceae) (Batta and Kavallieratos 2018), *Metarhizium* (Hypocreales: Clavicipitaceae) (Ashraf et al. 2017), *Isaria* (Hypocreales: Cordycipitaceae) (Zimmermann 2008), and *Beauveria* (Hypocreales: Cordycipitaceae) (Wakil et al. 2023a, b) have demonstrated remarkable efficacy in significantly decreasing insect populations. The generalist EPF *Metarhizium robertsii* (Metchnikoff) Sorokin (Hypocreales: Clavicipitaceae) exhibits the capacity to infect more than 200 different insect species, encompassing the orders Coleoptera, Lepidoptera, Hymenoptera, Diptera, Hemiptera, Dermaptera and Orthoptera (Brunner-Mendoza et al. 2019).

Lambda-cyhalothrin [RS-alpha-cyano-3-phenoxybenzyl 3-(2-chloro-3,32 V,3-trifluoropropenyl)-2,2,-dimethylcyclopropanecarboxylate] is a halogenated type II pyrethroid (Fetoui et al. 2015) and regarded as efficacious insecticide against a range of insect species, including storage pests (Moore et al. 2001; Jankov et al. 2013; Al-Sinjari and Al-Attar 2015; Shakoori et al. 2018; Feng et al. 2021). As a pyrethroid, lambda-cyhalothrin represents a synthetic chemical derivative of pyrethrins, which are naturally occurring insecticidal compounds found in the blossoms of *Tanacetum cinerariifolium* (Trevis.) Sch. Bip. (Asterales: Asteraceae) (He et al. 2008). Lambda-cyhalothrin disrupts nerve function in insects by binding to voltage-gated sodium channels. The binding prevents these channels from closing properly, leading to continuous nerve stimulation, tremors, and paralysis in affected insects (Shafer and Meyer 2004). Additionally, lambda-cyhalothrin can affect chloride and calcium channels, further interfering with nerve function. It is readily absorbed by biological tissues, penetrating the insect's cuticle, and causing rapid loss of muscular control, feeding cessation, and eventually death (Burr and Ray 2004; He et al. 2008). This insecticide has proven its efficacy against a range of stored-product pests, including *R. dominica, T. castaneum,* and *T. granarium* (Uddin and Ara 2006; Hamza 2018; Shakoori et al. 2018).

Within the framework of integrating insecticides to mitigate the challenges associated with the emergence of resistance and adverse environmental impacts (Gad et al. 2022), the current research endeavors to examine the effectiveness of the combination of lambda-cyhalothrin with *M. robertsii* or diatomaceous earth in laboratory and persistence trials, since there are no data available in the literature. The study focuses on three prominent storage pests known for their notoriety, in order to introduce a novel approach towards enhancing storage pest control methods.

## Materials and methods

### Insect culture

A population of unsexed and healthy, less than two weeks old adults of *R. dominica,* and *T. castaneum,* and < 24 h old for *T. granarium* was utilized (Wakil et al. 2022; Kavallieratos et al. 2022). These populations were sourced from long-standing cultures at the Microbial Control Laboratory, University of Agriculture, Faisalabad, Pakistan which had been isolated from exposure to pesticides, including phosphine for over twelve years. For cultiring *R. dominica* and *T. granarium*, environmental conditions of 30 °C, 65% RH, and a continuous 24-h darkness cycle were maintained. *Rhyzopertha dominica* and *T. granarium* were reared on whole wheat. In the case of *T. castaneum*, it was reared on a diet consisting of wheat flour and 5% brewer's yeast, under the same conditions (Kavallieratos et al. 2022; Wakil et al. 2022).

### Grains

In the experiment, whole soft wheat, *Triticum aestivum* L. var. Akbar 2019, was employed. The grains selected for use in the trials were free from any pest infestations, pesticides, or impurities. The moisture content of the wheat was determined at 11.20% using a calibrated Dicky-John moisture meter (Dickey-John Multigrain CAC II, Dickey-John Co., Auburn, IL, USA).

### Culturing of entomopathogenic fungi

The isolate of the entomopathogenic fungus *M. robertsii* (WG-56) was sourced from the inventory collection of the Microbial Control Laboratory, Department of Entomology, University of Agriculture, Faisalabad, Pakistan. To initiate the cultivation process, 100 mm in diameter Petri dishes were subjected to autoclaving. Subsequently, a culture of *M. robertsii* was spread onto Sabouraud dextrose agar (SDA) (Sigma-Aldrich Chemie GmbH, Taufkirchen, Germany). The Petri dishes were securely sealed with parafilm and then incubated using a MIR-254 incubator (Panasonic, Japan) at 25 °C, following a 14-h light/ 10-h dark photoperiod for 10 days (Wakil et al. 2023a, b). The resultant conidial layers were carefully harvested using a fine sterile scalpel and transferred into a 50 ml falcon tube containing 30 ml of a sterile 0.05% Tween 80 solution (Merk. Kenilworth, NJ, USA). The conidial suspension was introduced to a series of eight glass beads and vortexed for 5 min, using a Classic Vortex Mixer (VelpScientifica Srl, Usmate Velate, Italy). To achieve the desired concentration of *M. robertsii*, a Neubauer improved hemocytometer (Marienfeld, Lauda-Königshofen, Germany) was employed for microscopic observation under a microscope (Euromex BB.1152-PL, Arnhem, The Netherlands). For the assessment of conidial germination, 0.1 ml of the suspension was inoculated onto two petri dishes (60 mm ⌀) containing SDA + Yeast (SDAY) in a 20:1 ratio, which were then sealed with parafilm and incubated at 25 °C, following a 14:10-h (light: dark) photoperiod for 16 days. After incubation, sterile coverslips were placed on the dishes. Two separate dishes containing SDAY were used for the assessment of *M. robertsii*, with a total of 200 conidia examined. Conidia were considered germinated if a germ tube extending beyond the length of the conidium was observed (Inglis et al. 2012; Usman et al. 2020). This assessment was conducted using a Euromex BB.1152-PLi microscope (Arnhem, Netherlands) at a magnification of × 400. Before commencing the trials, a minimum conidial viability of 90% was maintained.

### Lambda-cyhalothrin

The lambda-cyhalothrin formulation (2.5% EC, Jaffer Group of Companies, Karachi, Pakistan) containing 2.5% of active ingredient (a.i.) was used in the experiments.

### Diatomaceous earth

The DE formulation (Protect-It, Hedley Technologies Inc., Mississauga, Ontario, Canada) was employed in the trials. The source of the DE is freshwater, and its composition comprises 89% amorphous silicon dioxide (SiO_2_), along with 1.7% Fe_2_O_3_, 4.0% Al_2_O_3_, less than 1% each of K_2_O and MgO, 1.4% CaO, and 3% moisture. The median particle size of the DE measures 10 μm, while it possesses a specific gravity (s.g.) of 2.2, a surface area of 35.7 m^2^/g, a pH value of 8, and a crystalline silica content of 0.1% (Wakil et al. 2010), which is significantly lower than the safe threshold of 1% for human health (Korunić 2013).

### Laboratory trials

The laboratory experiments comprised six distinct treatments plus a control group. These treatments encompassed the individual application of *M. robertsii* (Mr) at 1 × 10^7^ conidia/kg wheat, DE at 150 ppm (150 mg/kg wheat), and lambda-cyhalothrin (Lamb) at 1.25 mg/kg wheat, as well as their respective paired combinations, (Mr + Lamb, Mr + DE, Lamb + DE) at the same concentrations, plus the control group. One ml of a water solution containing 0.05% Tween 80 was administered to an additional 1 kg lot of wheat as a control. For each treatment, wheat lots of 1 kg were evenly distributed in thin layers across separate trays. Liquid formulations of *M. robertsii* and lambda-cyhalothrin were employed, whereas DE was utilized in the form of dust. Spraying was performed using distinct airbrushes (Master Multi-purpose Airbrush, USA), one dedicated to each treatment and a separate airbrush for control. One ml from each aqueous suspension (Mr, Lamb or control) was administered to one kg of wheat. Subsequently, the treated grains were transferred into separate 3-l glass jars and manually agitated for 10 min to achieve a uniform dispersion of conidia, the insecticide or Tween 80 throughout the grain mass. Regarding the single application of DE, 1 kg of treated wheat was conveyed into a 3-L glass jar and shaken as previously described. In the case of the paired treatment using aqueous suspensions, 1 ml of each aqueous suspension (Mr or Lamb) was applied to 1 kg of wheat using a separate airbrush. Regarding the paired treatments that involved a combination of different states of the materials, including both aqueous (Mr or Lamb) and dust (DE) applications, each 1 ml of aqueous suspension was initially and individually applied, followed by the application of DE. Subsequently, the treated wheat was transferred to 3-L glass jars where it was shaken as previously outlined. Three 100-g wheat samples were extracted from each jar, including the control jar, and transferred into individual plastic vials (11 cm height, 6.5 cm diameter). The precise weight of 100 g of wheat was measured using the ELB 300 Shimadzu compact balance (Kyoto, Japan). The vial lids were equipped with a central 15 mm diameter hole, covered with gauze to ensure adequate aeration within the vial. To deter the escape of insects, the inner upper surfaces of the vials were treated with a 60% aqueous dispersion of polytetrafluoroethylene (Sigma-Aldrich Chemie GmbH, Taufkirchen, Germany). Thereafter, 50 adults of *R. dominica* were released into each vial and preserved at 20 °C and 65% RH. Adult mortality was assessed 7 and 14 days following the treatment application using a Leica Wild M3B stereomicroscope (Heerbrugg, Switzerland) at a magnification of × 40. For each exposure interval separate series of vials were prepared. After the experimental period, the insects that had been subjected to testing were extracted from the wheat. The wheat was then placed back into vials and returned to the incubators, maintaining the previously specified conditions to document offspring production. Progeny production was documented at 62 days after the last mortality count (14 days) (Wakil et al. 2022). The same procedure was conducted for *T. castaneum* and *T. granarium* for which progeny was recorded at 62 and 46 days, respectively (Wakil et al. 2022). In the case of *R. dominica*, progeny determination exclusively considered adult specimens, as the immature life stages of this species are confined within the grain. For the remaining species, both immatures and adults were documented as progeny. The entire experimental procedure was replicated twice using a new series of jars, vials, and insect specimens for each replication.

### Persistence trials

Following a 7-day initial exposure period of the insects to treated wheat, an evaluation of the single and paired treatments applied on wheat was conducted over 120 days by performing five bioassays at intervals of 30 days (i.e., at 0, 30, 60, 90, and 120 days) under controlled conditions of 65% RH and 30 °C, following the same procedures as in the laboratory bioassays. The term "day 0" signifies the initial day subsequent to the 7-day exposure period to the wheat treated with insecticides. Throughout the entire course of the experiment, three sets of 100 g of wheat, each treated with the corresponding control agents, were securely stored in sealed containers under the identical temperature and RH conditions previously specified. The entire procedure was conducted on three separate occasions, with each iteration entailing the creation of new wheat batches, vials, and insects. The persistence trials were replicated once more, following a 14-day initial exposure of the insects to the treated wheat. For each specific interval, distinct containers were employed.

### Data analysis in laboratory trials

Mortality data were adjusted using Abbott's equation (Abbott 1925) and subjected to a log transformation of (x + 1) to ensure a normalized variance before proceeding with statistical analysis (Zar 2010; Scheff and Arthur 2018). The mortality data obtained from the laboratory trials underwent a two-way analysis of variance (ANOVA). In this analysis, the main effects were the exposure interval and treatment, while the response variable was mortality. Interactions among all main effects were taken into account. In the case of evaluating offspring in laboratory bioassays, a two-way ANOVA was conducted, where the main effects included treatment and insect species, and the response variable was the count of individuals in the succeeding generation. Progeny of the controls was also incorporated into the analysis. All statistical analyses were conducted using Minitab software (Minitab 2017). To enable the comparison of mortality rates and offspring, the Tukey–Kramer (HSD) test was employed at a significance level of α = 0.05 (Sokal and Rohlf 1995).

### Data analysis in persistence trials

Mortality data were adjusted with Abbott's equation (Abbott 1925). Data were log (x + 1) transformed prior to the conduction of the statistical analysis to ensure a normalized variance (Zar 2010; Scheff and Arthur 2018). The mortality data obtained from the persistence trials were subjected to two two-way ANOVA, with the storage period and treatment as the main effects, and mortality as the response variable. The associated interaction of the main effects was incorporated into the analysis. When assessing offspring in persistence bioassays, another two-way ANOVA was conducted, with the main effects being treatment and storage period, and the count of emerging individuals as the response variable. The analysis also took into account interactions involving these main effects. To facilitate comparisons of progeny and mortality means, the Tukey–Kramer (HSD) test was employed at a significance level of α = 0.05 (Sokal and Rohlf 1995). All statistical analyses were conducted using Minitab 2017 (Minitab 2017).

## Results

### Adult mortality and progeny production in laboratory trials

All main effects were significant for the mortality of all three insect species tested, while their interaction (treatment × exposure) was significant only for *T. granarium* and *R. dominica* (Table 1). Across all insect species, mortality rates exhibited an upward trend as the duration of exposure increased (Table 2). While there were no instances of complete (100%) mortality, significantly higher mortality rates were documented in paired treatments when compared to single treatments for all insect species. On most occasions, DE + Lamb caused significantly higher mortalities compared to the remaining paired treatments across all insect species and exposure intervals, apart from the mortality of *T. castaneum* at 7 days of exposure, where the Mr + Lamb and DE + Lamb treatments did not exhibit a significant difference. Differences in mortalities resulting from the Mr + Lamb and Mr + DE treatments were significant at 14 days post-exposure for all the pest species tested and for *T. castaneum* at 7 days of exposure. Differences in mortalities caused by the Mr + Lamb and Mr + DE treatments remained insignificant for the remaining insect species at 7 days after exposure. Among the paired treatments, DE + Lamb proved to be the most effective, exhibiting 64.43%, 58.30%, and 72.50% mortalities for *T. castaneum, T. granarium*, and *R. dominica*, respectively at 7 days post-exposure. The DE + Lamb treatment remained the most efficient at 14 days of exposure, demonstrating 83.37%, 76.59%, and 94.55% mortalities for the aforementioned species, respectively. Following this combination, Mr + Lamb induced 57.29%, 49.14%, and 63.34% mortality to *T. castaneum, T. granarium*, and *R. dominica*, respectively at 7 days of exposure, and 71.52%, 65.38%, and 77.62% at 14 days post-exposure. The joint action of Mr + DE caused 48.13%, 44.37%, and 58.62% mortality at 7 days of exposure, and 62.66%, 53.21%, and 69.47% mortality at 14 days post-exposure to *T. castaneum, T. granarium*, and *R. dominica*, respectively. The single treatments in descending order of effectiveness were Lamb, DE, and Mr. *Tribolium castaneum* exhibited 38.26% and 49.12% mortality at 7 and 14 days post-exposure, respectively, when exposed to Lamb, while the remaining single treatments resulted in less than 39% and 28% mortality at 14 and 7 days of exposure, respectively. Single treatments resulted in less than 42% mortality for *T. granarium* at all exposure intervals, with the highest mortality record observed at 14 days post-exposure when exposed to Lamb (41.66%). For *R. dominica*, Lamb resulted in 57.24% and 46.10% mortality at 14- and 7-days post-exposure, followed by DE (46.10% and 36.29%, respectively), while Mr caused less than 39% mortality across all exposure intervals. Overall, regardless of the treatment and exposure interval, *R. dominica* was the most susceptible, followed by *T. castaneum* and *T. granarium*.

**Table 1:** ANOVA parameters for adult mortality of *T. castaneum, T. granarium,* and *R. dominica* on wheat treated with *M. robertsii*, lambda-cyhalothrin, and diatomaceous earth alone and in their respective paired combinations in laboratory trials (total *df* = 71)

		Insect species					
		*T. castaneum*		*T. granarium*		*R. dominica*	
Effect	*df*	*F*	*P*	*F*	*P*	*F*	*P*
Treatment	5	200.7	< 0.01	237.5	< 0.01	198.6	< 0.01
Exposure interval	1	152.2	< 0.01	173.9	< 0.01	140.9	< 0.01
Treatment × exposure interval	5	2.2	0.06	3.9	< 0.01	3.3	0.01

**Table 2:** Mean mortality (% ± SE) of *T. castaneum, T. granarium,* and *R. dominica* adults after a 7- and 14-day exposure on wheat treated with *M. robertsii* (Mr) at 1 × 10^7^ conidia/kg wheat, DE at 150 ppm (150 mg/kg wheat), and lambda-cyhalothrin (Lamb) at 1.25 mg/kg wheat, and their respective paired combinations, Mr + Lamb, Mr + DE, Lamb + DE at the same concentrations, in laboratory trials

		Days post-exposure			
Insect species	Treatment	7	14	*F*	*P*
*T. castaneum*	Mr	23.41 ± 1.43 Bd	31.51 ± 1.06 Ae	20.5	< 0.01
	Lamb	38.26 ± 2.01 Bc	49.12 ± 1.36 Ad	19.9	< 0.01
	DE	27.45 ± 1.92 Bd	38.27 ± 2.74 Ae	10.4	< 0.01
	Mr + DE	48.13 ± 0.80 Bb	62.66 ± 2.27 Ac	36.1	< 0.01
	DE + Lamb	64.43 ± 2.35 Ba	83.37 ± 2.05 Aa	36.8	< 0.01
	Mr + Lamb	57.29 ± 1.52 Ba	71.52 ± 1.06 Ab	58.3	< 0.01
	*F*	87.6	114.0		
	*P*	< 0.01	< 0.01		
*T. granarium*	Mr	18.64 ± 1.09 Bd	29.47 ± 1.01 Ae	52.6	< 0.01
	Lamb	32.49 ± 2.91 Bc	41.66 ± 1.45 Ad	7.9	< 0.01
	DE	24.39 ± 1.33 Bd	32.18 ± 1.11 Ae	20.2	< 0.01
	Mr + DE	44.37 ± 1.72 Bb	53.21 ± 0.71 Ac	22.4	< 0.01
	DE + Lamb	58.30 ± 1.26 Ba	76.59 ± 1.59 Aa	81.0	< 0.01
	Mr + Lamb	49.14 ± 1.16 Bb	65.38 ± 2.07 Ab	46.8	< 0.01
	*F*	80.4	181.0		
	*P*	< 0.01	< 0.01		
*R. dominica*	Mr	29.14 ± 0.59 Bd	38.29 ± 1.10 Af	53.7	< 0.01
	Lamb	46.10 ± 2.31 Bc	57.24 ± 2.06 Ad	12.9	< 0.01
	DE	36.29 ± 2.18 Bd	46.10 ± 1.68 Ae	12.6	< 0.01
	Mr + DE	58.62 ± 1.47 Bb	69.47 ± 1.77 Ac	22.1	< 0.01
	DE + Lamb	72.50 ± 1.97 Ba	94.55 ± 1.87 Aa	65.5	< 0.01
	Mr + Lamb	63.34 ± 2.64 Bb	77.62 ± 1.97 Ab	18.8	< 0.01
	*F*	70.9	138.0		
	*P*	< 0.01	< 0.01		

Within each row, means followed by the same uppercase letter are not significantly different; *df* = 1, 11, Tukey–Kramer (HSD) test at *P* = 0.05. For each species, within each column, means followed by the same lowercase letter are not significantly different; *df* = 5, 35, Tukey–Kramer (HSD) test at *P* = 0.05

Concerning progeny, all main effects and associated interaction were significant (Table 3). The minimum progeny production was recorded for DE + Lamb, although not suppressed, followed by Mr + Lamb, Mr + DE, Lamb, DΕ, and Mr among all insect species (Table 4). The joint treatments resulted in significantly lower offspring numbers compared to the single ones across all insect species tested. Among the paired treatments, DE + Lamb demonstrated significantly lower progeny than the remaining treatments for all insect species apart from *T. castaneum*. *Tribolium castaneum* demonstrated 18.48, 25.21, and 29.61 individuals/vial when treated with DE + Lamb, Mr + Lamb, and Mr + DE, respectively. The offspring production of *T. granarium* reached 23.88, 31.40, and 35.23 individuals/vial for DE + Lamb, Mr + Lamb, and Mr + DE, respectively, while for *R. dominica* these treatments resulted in 7.81, 16.70, and 25.51 individuals/vial, respectively. Regarding the single treatments, Lamb caused significantly lower progeny than the remaining treatments across all insect species, resulting in 38.73, 47.78, and 31.63 individuals/vial for *T. castaneum, T. granarium*, and *R. dominica*, respectively. The remaining single treatments caused more than 46, 61, and 52 individuals/vial for *T. castaneum, T. granarium*, and *R. dominica*, respectively. Overall, the lowest progeny was observed for *R. dominica*, followed by *T. castaneum* and *T. granarium* across all paired and single treatments.

**Table 3:** ANOVA parameters for progeny production of *T. castaneum, T. granarium,* and *R. dominica* on wheat treated with *M. robertsii*, lambda-cyhalothrin, and diatomaceous earth alone and in their respective paired combinations in laboratory trials (total *df* = 125)

Effect	*df*	*F*	*P*
Treatment	6	1111.7	< 0.01
Insect species	2	112.4	< 0.01
Treatment × insect species	12	17.8	< 0.01

**Table 4:** Mean progeny number (± SE) of *T. castaneum, T. granarium,* and *R. dominica* individuals/vial, following a 62-, 46-, and 62-day period, respectively, on wheat treated with *M. robertsii* (Mr) at 1 × 10^7^ conidia/kg wheat, DE at 150 ppm (150 mg/kg wheat), and lambda-cyhalothrin (Lamb) at 1.25 mg/kg wheat, and their respective paired combinations, Mr + Lamb, Mr + DE, Lamb + DE at the same concentrations, in laboratory trials

	Treatment								
Insect species	Mr	Lamb	DE	Mr + DE	DE + Lamb	Mr + Lamb	Control	*F*	*P*
*T. castaneum*	49.58 ± 1.32 Cb	38.73 ± 1.46 Bc	46.36 ± 2.55 Bb	29.61 ± 1.30 ABd	18.48 ± 1.41 Be	25.21 ± 1.41 Bde	93.41 ± 0.72 Ca	260.0	< 0.01
*T. granarium*	72.50 ± 0.63 Ab	47.78 ± 1.40 Ad	61.58 ± 1.92 Ac	35.23 ± 1.93 Ae	23.88 ± 1.49 Af	31.40 ± 0.44 Ae	105.60 ± 1.60 Ba	383.0	< 0.01
*R. dominica*	56.28 ± 1.34 Bb	31.63 ± 0.92 Cc	52.60 ± 0.78 Bb	25.51 ± 1.58 Bc	7.81 ± 1.29 Ce	16.70 ± 1.17 Cd	114.50 ± 3.04 Aa	493.0	< 0.01
*F*	101.0	39.4	16.2	9.0	33.9	45.7	27.1		
*P*	< 0.01	< 0.01	< 0.01	< 0.01	< 0.01	< 0.01	< 0.01		

Within each row, means followed by the same lowercase letter are not significantly different; *df* = 6, 41, Tukey–Kramer (HSD test at *P* = 0.05. Within each column, means followed by the same uppercase letter are not significantly different; *df* = 2, 17, Tukey–Kramer (HSD) test at *P* = 0.05

### Adult mortality and progeny production in persistence trials

All main effects were significant for the mortality of all three insect species tested, while their interaction (treatment × storage period) was significant only for *T. castaneum* and *R. dominica* when initially exposed to treatments for 7 days (Table 5). In persistence trials after an initial 7-day exposure, mortality rates decreased over time among all insect species and treatments, while no complete mortality was documented (Table 6). Higher mortality rates were documented for paired treatments compared to single treatments for all insect species, although the differences in mortalities between joint and single treatments were not always significant. Among the paired treatments, DE + Lamb had the strongest effect against all insect species, followed by Mr + Lamb, and Mr + DE whilst differences in mortalities were significant only at 0 days of storage. The highest mortality for *T. castaneum* was exhibited for DE + Lamb (58.03%), followed by Mr + Lamb (49.50%), and Mr + DE (37.87%) at 0 days of storage, while it decreased with time reaching 32.76%, 26.60%, and 23.20% at 120 days of storage for the aforementioned treatments, respectively. *Trogoderma granarium* demonstrated 51.19%, 42.34%, and 32.41% mortality at 0 days of storage when treated with DE + Lamb, Mr + Lamb, and Mr + DE, respectively, whereas mortalities for all paired treatments were less than 31% at 120 days of storage. The DE + Lamb treatment caused 63.81% mortality to *R. dominica* at 0 days of storage, followed by Mr + Lamb (52.23%), and Mr + DE (44.35%). Mortality gradually declined to 37.18%, 35.14%, and 26.61% 120 days later for the aforementioned paired treatments, respectively. Insignificant differences in the mortality of *R. dominica* and *T. castaneum* were observed between 0 and 30 days of storage when treated with DE + Lamb, while it was significant for *T. granarium.* Over the storage period, individual treatments showed a decrease, with variations in mortalities not consistently reaching significance across all species and storage durations. *Tribolium castaneum* exhibited 28.66% mortality when treated with Lamb, whereas the remaining single treatments demonstrated less than 20% mortalities. At the final count of the persistence trials *T. castaneum* displayed less than 9% mortality, however, higher mortality was observed for DE (8.51%), followed by Mr (7.83%), and Lamb (3.74%). For *T. granarium*, Lamb resulted in 25.24% mortality at the initiation of the experiment, followed by DE (17.40%), and Mr (13.62%). At 120 days of storage, DE caused *T. granarium* 4.42% mortality, while the remaining single treatments had no effect on mortality (0.0%). Concerning *R. dominica*, at 0 days of storage, Lamb caused the highest observed mortality among the single treatments (34.44%), followed by DE (27.28%), and Mr (23.55%), while the same treatments resulted in 9.19%, 15.71%, and 11.59% mortalities, respectively. Overall, *R. dominica* was the most susceptible species, followed by *T. castaneum*, and *T. granarium*.

**Table 5:** ANOVA parameters for adult mortality of *T. castaneum, T. granarium,* and *R. dominica* after a 7-day exposure on wheat treated with *M. robertsii*, lambda-cyhalothrin, and diatomaceous earth alone and in their respective paired combinations in persistence trials (total *df* = 179)

		Insect species					
		*T. castaneum*		*T. granarium*		*R. dominica*	
Effect	*df*	*F*	*P*	*F*	*P*	*F*	*P*
Treatment	5	308.0	< 0.01	256.7	< 0.01	324.6	< 0.01
Storage period	4	103.8	< 0.01	89.2	< 0.01	117.8	< 0.01
Treatment × storage period	20	3.0	< 0.01	1.5	0.08	3.4	< 0.01

**Table 6:** Mean mortality (% ± SE) of *T. castaneum, T. granarium,* and *R. dominica* adults after a 7-day exposure on wheat treated with *M. robertsii* (Mr) at 1 × 10^7^ conidia/kg wheat, DE at 150 ppm (150 mg/kg wheat), and lambda-cyhalothrin (Lamb) at 1.25 mg/kg wheat, and their respective paired combinations, Mr + Lamb, Mr + DE, Lamb + DE at the same concentrations, in five storage periods carried out from 0 to 120 days after treatment in persistence trials

		Storage period (days)						
Insect species	Treatment	0	30	60	90	120	*F*	*P*
*T. castaneum*	Ma	17.73 ± 1.21 Ae	13.64 ± 1.34 Abd	11.59 ± 1.34 BCc	8.17 ± 1.16 Cc	7.83 ± 0.96 Cc	11.4	< 0.01
	Lamb	28.66 ± 1.72 Ad	24.58 ± 0.95 Ac	17.75 ± 0.45 Bc	10.58 ± 1.10 Cc	3.74 ± 1.10 Dc	78.4	< 0.01
	DE	19.79 ± 1.44 Ae	18.76 ± 1.60 Acd	16.39 ± 0.78 Ac	13.65 ± 1.55 ABc	8.51 ± 2.25 Bc	8.0	< 0.01
	Ma + DE	37.87 ± 1.76 Ac	34.14 ± 1.60 Abb	31.39 ± 2.71 ABCb	28.33 ± 2.39 BCb	23.20 ± 0.85 Cb	6.2	< 0.01
	DE + Lamb	58.03 ± 1.16 Aa	47.45 ± 1.86 Ba	43.35 ± 2.10 BCa	38.22 ± 2.81 CDa	32.76 ± 2.35 Da	20.3	< 0.01
	Ma + Lamb	49.50 ± 2.00 Ab	43.35 ± 1.57 Aba	37.18 ± 1.14 BCab	33.44 ± 1.13 Cab	26.60 ± 1.54 Dab	34.0	< 0.01
	*F*	105.0	80.4	62.7	38.5	53.6		
	*P*	< 0.01	< 0.01	< 0.01	< 0.01	< 0.01		
*T. granarium*	Ma	13.62 ± 1.85 Ad	11.25 ± 1.13 Abd	7.50 ± 1.24 Bd	1.36 ± 0.68 Cb	0.00 ± 0.00 Cc	26.4	< 0.01
	Lamb	25.24 ± 2.25 Ac	19.45 ± 0.68 Bc	16.37 ± 0.71 Bc	8.51 ± 1.21 Cb	0.00 ± 0.00 Dc	64.1	< 0.01
	DE	17.40 ± 1.55 Ad	12.62 ± 1.43 ABcd	10.58 ± 1.22 ABcd	7.50 ± 2.33 Bb	4.42 ± 2.85 Bc	6.3	< 0.01
	Ma + DE	32.41 ± 2.18 Ac	31.40 ± 2.16 Abb	25.26 ± 1.76 ABCb	23.53 ± 3.13 BCa	18.43 ± 0.53 Cb	7.4	< 0.01
	DE + Lamb	51.19 ± 1.19 Aa	44.36 ± 2.65 Aba	39.61 ± 1.66 BCa	32.74 ± 2.32 Ca	30.38 ± 1.73 Ca	11.0	< 0.01
	Ma + Lamb	42.34 ± 1.47 Ab	37.19 ± 0.92 Abb	35.48 ± 1.31 Ba	27.31 ± 1.96 Ca	21.82 ± 2.05 Cb	26.1	< 0.01
	*F*	65.5	68.0	93.6	24.3	62.7		
	*P*	< 0.01	< 0.01	< 0.01	< 0.01	< 0.01		
*R. dominica*	Ma	23.55 ± 1.16 Ae	21.49 ± 1.36 Abd	17.40 ± 0.86 BCd	16.38 ± 0.91 CDe	11.59 ± 1.53 Dc	15.2	< 0.01
	Lamb	34.44 ± 1.77 Ad	30.38 ± 1.49 Abc	28.30 ± 2.45 ABbc	21.15 ± 0.97 Bd	9.19 ± 1.71 Cc	33.0	< 0.01
	DE	27.28 ± 1.67 Ae	25.58 ± 1.78 ABcd	21.49 ± 1.34 BCcd	18.43 ± 0.53 Cde	15.71 ± 1.28 Cc	12.0	< 0.01
	Ma + DE	44.35 ± 1.29 Ac	37.54 ± 0.84 Bb	32.75 ± 1.55 BCb	31.39 ± 1.24 CDc	26.61 ± 1.39 Db	27.2	< 0.01
	DE + Lamb	63.81 ± 1.98 Aa	54.17 ± 1.55 Ba	48.11 ± 1.59 Ba	46.41 ± 0.82 Ba	37.18 ± 1.14 Ca	42.4	< 0.01
	Ma + Lamb	52.23 ± 1.64 Ab	49.83 ± 2.69 Aa	47.11 ± 2.24 Aba	38.57 ± 1.44 BCb	35.14 ± 2.58 Ca	11.4	< 0.01
	*F*	91.3	51.9	53.2	137.0	52.1		
	*P*	< 0.01	< 0.01	< 0.01	< 0.01	< 0.01		

Within each row, means followed by the same uppercase letter are not significantly different; *df* = 4, 29, Tukey–Kramer (HSD) test at *P* = 0.05. For each species, within each column, means followed by the same lowercase letter are not significantly different; *df* = 5, 35, Tukey–Kramer (HSD) test at *P* = 0.05

Significant main effects were observed for the mortality of all three insect species tested after 14 days of initial exposure to the treated wheat (Table 7). However, the interaction between treatment and storage period was only significant for *T. castaneum*. Following an initial 14-day exposure, mortality rates decreased over time for all insect species and treatments, with no cases of complete mortality observed (Table 8). Significantly higher mortality rates were documented for paired treatments compared to single treatments for all insect species. Among paired treatments, differences were mostly significant at 0, 30, 60, and 90 days of exposure, with DE + Lamb being the most effective treatment, followed by Mr + Lamb, and Mr + DE. *Tribolium castaneum* demonstrated 74.40% mortality when treated with DE + Lamb, while Mr + Lamb and Mr + DE resulted in 65.85% and 47.76% mortality at day 0, respectively. At the final count, the mortality of *T. castaneum* reached 36.52% for DE + Lamb, followed by 31.39% for Mr + Lamb and 26.29% for Mr + DE. The highest mortality rate for *T. granarium* (67.56%) was achieved at day 0 when exposed to DE + Lamb, followed by Mr + Lamb (56.31%) and Mr + DE (40.61%). After 120 days T*. granarium* exhibited 41.64%, 35.82%, and 22.16% mortality when exposed to DE + Lamb, Mr + Lamb, and Mr + DE, respectively. The DE + Lamb treatment caused *R. dominica* 86.67% mortality at the initiation of the experiment, whereas Mr + Lamb and Mr + DE resulted to 73.37% and 58.71% mortality, respectively. At the final count, *R. dominica* demonstrated 58.36%, 45.38%, and 39.25% mortality for the aforementioned treatments, respectively. Insignificant differences in the mortality of *T. castaneum* were observed between 0 and 60 days of storage when treated with DE + Lamb, as well as for *R. dominica* between 0 and 30 days of storage, while differences were significant for *T. granarium.* During the storage period, individual treatments exhibited a decline, with disparities in mortality not consistently attaining statistical significance across all species and storage periods. At day 0, *T. castaneum* reached 36.50%, 28.32%, and 25.56% mortality for Lamb, DE, and Mr, respectively. However, after 120 days of storage, DE caused *T. castaneum* 14.67% mortality, followed by Lamb (10.55%), and Mr (7.51%). This alternating trend was consistent with the remaining species. *Trogoderma granarium* reached 34.12%, 28.32%, and 23.18% mortality at 0 days of storage when treated with Lamb, DE, and Mr, respectively, but demonstrated 14.67%, 10.55%, and 7.51% mortality rates 120 days later when treated with DE, Lamb, and Mr, respectively. Concerning *R. dominica*, Lamb resulted in 42.65% mortality at day 0, followed by DE (35.83%), and Mr (31.38%), whereas at 120 days of storage, DE, Lamb, and Mr caused 19.11%, 14.67%, and 11.27% mortality, respectively. In general, *R. dominica* exhibited the highest susceptibility, followed by *T. castaneum*, and *T. granarium*.

**Table 7:** ANOVA parameters for adult mortality of *T. castaneum, T. granarium,* and *R. dominica* after a 14-day exposure on wheat treated with *M. robertsii*, lambda-cyhalothrin, and diatomaceous earth alone and in their respective paired combinations in persistence trials (total *df* = 179)

		Insect species					
		*T. castaneum*		*T. granarium*		*R. dominica*	
Effect	*df*	*F*	*P*	*F*	*P*	*F*	*P*
Treatment	5	585.6	< 0.01	317.3	< 0.01	663.2	< 0.01
Storage period	4	228.2	< 0.01	98.1	< 0.01	167.7	< 0.01
Treatment × storage period	20	6.4	< 0.01	1.1	0.39	2.0	0.1

**Table 8:** Mean mortality (% ± SE) of *T. castaneum, T. granarium,* and *R. dominica* adults after a 14-day exposure on wheat treated with *M. robertsii* (Mr) at 1 × 10^7^ conidia/kg wheat, DE at 150 ppm (150 mg/kg wheat), and lambda-cyhalothrin (Lamb) at 1.25 mg/kg wheat, and their respective paired combinations, Mr + Lamb, Mr + DE, Lamb + DE at the same concentrations, in five storage periods carried out from 0 to 120 days after treatment in persistence trials

		Storage period (days)						
Insect species	Treatment	0	30	60	90	120	*F*	*P*
*T. castaneum*	Ma	25.56 ± 2.10 Ad	21.14 ± 1.42 ABe	18.41 ± 1.45 Bd	11.59 ± 1.34 Ce	7.51 ± 0.86 Cd	23.7	< 0.01
	Lamb	36.50 ± 2.57 Ac	29.36 ± 1.68 ABd	25.60 ± 1.84 Bd	16.71 ± 1.08 Cde	10.55 ± 1.68 Ccd	31.1	< 0.01
	DE	28.32 ± 0.73 Acd	25.26 ± 2.72 Ade	21.16 ± 0.71 Bd	19.45 ± 0.89 Bd	14.67 ± 1.10 Cc	37.9	< 0.01
	Ma + DE	47.76 ± 1.01 Ab	44.36 ± 1.33 ABc	38.21 ± 2.11 Bc	31.41 ± 1.60 Cc	26.29 ± 1.64 Cb	31.3	< 0.01
	DE + Lamb	74.40 ± 1.54 Aa	70.30 ± 2.27 Aa	67.24 ± 2.39 Aa	53.23 ± 1.12 Ba	36.52 ± 1.34 Ca	73.3	< 0.01
	Ma + Lamb	65.85 ± 2.96 Aa	58.36 ± 1.24 ABb	52.89 ± 1.18 BCb	45.38 ± 1.45 Cb	31.39 ± 1.92 Dab	49.4	< 0.01
	*F*	102.0	168.0	129.0	173.0	65.0		
	*P*	< 0.01	< 0.01	< 0.01	< 0.01	< 0.01		
*T. granarium*	Ma	23.18 ± 1.75 Ae	16.70 ± 1.59 Bd	13.64 ± 0.99 BCe	8.52 ± 1.21 CDd	3.42 ± 0.87 Dd	32.3	< 0.01
	Lamb	34.12 ± 2.47 Acd	25.60 ± 2.42 ABcd	22.50 ± 2.14 BCd	15.35 ± 0.86 CDc	6.46 ± 1.70 Dcd	20.9	< 0.01
	DE	28.32 ± 0.78d Ae	24.58 ± 0.79 Ad	19.44 ± 0.83 Bde	16.70 ± 1.19 Bc	11.27 ± 1.28 Cc	44.0	< 0.01
	Ma + DE	40.61 ± 0.77 Ac	37.54 ± 0.99 Abc	36.17 ± 0.98 Ac	28.67 ± 1.39 Bb	22.16 ± 2.29 Cb	28.7	< 0.01
	DE + Lamb	67.56 ± ± 2.56 Aa	64.14 ± 2.00 ABa	55.26 ± 2.24 BCa	47.76 ± 2.60 CDa	41.64 ± 0.91 Da	14.2	< 0.01
	Ma + Lamb	56.31 ± 1.71 Ab	49.13 ± 2.05 ABb	46.74 ± 1.72 ABCb	42.31 ± 1.23 BCa	35.82 ± 2.12 Ca	7.8	< 0.01
	*F*	54.8	36.6	107.0	108.0	94.6		
	*P*	< 0.01	< 0.01	< 0.01	< 0.01	< 0.01		
*R. dominica*	Ma	31.38 ± 1.29 Ae	28.31 ± 1.74 Ae	21.14 ± 1.42 Be	18.41 ± 1.14 Be	11.27 ± 1.57 Ce	30.4	< 0.01
	Lamb	42.65 ± 2.14 Ad	37.20 ± 2.56 ABd	32.76 ± 1.90 ABd	26.28 ± 1.11 Bd	14.67 ± 0.81 Cde	16.4	< 0.01
	DE	35.83 ± 1.24 Ade	33.44 ± 1.60 Ade	24.56 ± 1.71 Be	21.49 ± 1.00 Bde	19.11 ± 0.71 Bd	31.6	< 0.01
	Ma + DE	58.71 ± 1.13 Ac	56.30 ± 0.92 Ac	48.10 ± 2.13 Bc	43.67 ± 1.91 BCc	39.25 ± 1.22 Cc	28.7	< 0.01
	DE + Lamb	86.67 ± 2.53 Aa	82.24 ± 1.04 Aa	71.67 ± 2.12 Ba	64.17 ± 1.77 BCa	58.36 ± 1.13 Ca	42.9	< 0.01
	Ma + Lamb	73.37 ± 1.21 Ab	65.52 ± 1.78 Bb	63.12 ± 1.28 Bb	56.29 ± 1.58 Cb	45.38 ± 1.45 Db	50.6	< 0.01
	*F*	98.5	114.0	135.0	172.0	255.0		
	*P*	< 0.01	< 0.01	< 0.01	< 0.01	< 0.01		

Within each row, means followed by the same uppercase letter are not significantly different; *df* = 4, 29, Tukey–Kramer (HSD) test at *P* = 0.05. For each species, within each column, means followed by the same lowercase letter are not significantly different; *df* = 5, 35, Tukey–Kramer (HSD) test at *P* = 0.05

Concerning progeny emergence, all the main effects and associated interaction were significant for all insect species tested (Table 9). The minimal progeny production was documented in the case of DE + Lamb, although it was not entirely suppressed (Table 10). Insignificant differences in the progeny number of *T. castaneum* and *R. dominica* were observed between 0 and 30 days of storage when treated with DE + Lamb, while it was significant for *T. granarium*. Subsequently, Mr + Lamb, Mr + DE, Lamb, DE, and Mr followed in descending order across all insect species. The paired treatments resulted in significantly reduced offspring numbers when compared to the individual treatments across all insect species and storage periods under examination. *Tribolium castaneum* demonstrated 21.58, 34.20, and 38.23 individuals/vial at the initiation of the experiment when treated with DE + Lamb, Mr + Lamb, and Mr + DE, respectively, whereas single treatments resulted in more than 46 individuals/vial. After 120 days, progeny numbers increased, reaching more than 55, and 78 individuals/vial in paired and single treatments, respectively. Regarding *T. granarium*, DE + Lamb, Mr + Lamb, and Mr + DE resulted in 38.41, 41.16, and 52.71 individuals/vial, respectively. Single treatments resulted in more than 71 T*. granarium* individuals/vial. At 120 days of storage, *T. granarium* progeny was higher than 79 and 115 individuals/vial for paired and single treatments, respectively. The DE + Lamb, Mr + Lamb, and Mr + DE treatments elicited 18.56, 21.60, and 27.83 *R. dominica* individuals/vial at day 0, respectively, while single treatments resulted in more than 36 individuals/vial. At the final count, paired and single treatments demonstrated more than 46, and 63 individuals/vial, respectively. Overall, the lowest progeny was observed for *R. dominica*, followed by *T. castaneum* and *T. granarium* across all paired and single treatments.

**Table 9:** ANOVA parameters for progeny production of *T. castaneum, T. granarium,* and *R. dominica* on wheat treated with *M. robertsii*, lambda-cyhalothrin, and diatomaceous earth alone and in their respective paired combinations in persistence trials (total *df* = 209)

		Insect species					
		*T. castaneum*		*T. granarium*		*R. dominica*	
Effect	*df*	*F*	*P*	*F*	*P*	*F*	*P*
Treatment	6	1529.1	< 0.01	2092.1	< 0.01	2276.4	< 0.01
Storage period	4	510.5	< 0.01	1254.5	< 0.01	844.3	< 0.01
Treatment × storage period	24	3.7	< 0.01	3.3	< 0.01	7.0	< 0.01

**Table 10:** Mean progeny number (± SE) of *T. castaneum, T. granarium,* and *R. dominica* individuals/vial on wheat treated with *M. robertsii* (Mr) at 1 × 10^7^ conidia/kg wheat, DE at 150 ppm (150 mg/kg wheat), and lambda-cyhalothrin (Lamb) at 1.25 mg/kg wheat, and their respective paired combinations, Mr + Lamb, Mr + DE, Lamb + DE at the same concentrations, in five storage periods carried out from 0 to 120 days after treatment in persistence trials

		Storage period (days)						
Insect species	Treatment	0	30	60	90	120	*F*	*P*
*T. castaneum*	Ma	62.40 ± 0.80 Eb	69.11 ± 1.38 Db	76.20 ± 1.57 Cb	87.28 ± 1.16 Bb	95.61 ± 1.36 Ab	109.0	< 0.01
	Lamb	46.15 ± 1.22 Ec	53.21 ± 1.24 Dc	59.10 ± 1.14 Cc	66.45 ± 1.34 Bc	78.26 ± 1.77 Acd	81.9	< 0.01
	DE	57.75 ± 1.65 Db	64.00 ± 0.91 Cb	72.06 ± 1.15 Bb	69.35 ± 1.43 Bc	82.40 ± 0.60 Ac	57.9	< 0.01
	Ma + DE	38.23 ± 1.02 Ed	45.38 ± 1.23 Dd	52.36 ± 1.83 Cd	58.31 ± 1.06 Bd	74.20 ± 1.28 Ad	108.0	< 0.01
	DE + Lamb	21.58 ± 0.59 De	28.53 ± 1.48 De	35.75 ± 1.21 Cf	42.25 ± 3.06 Be	55.50 ± 1.06 Af	61.7	< 0.01
	Ma + Lamb	34.20 ± 0.76 Dd	41.16 ± 2.23 CDd	43.65 ± 2.01 Ce	56.58 ± 1.47 Bd	63.83 ± 1.71 Ae	49.2	< 0.01
	Control	96.30 ± 1.55 Ca	108.87 ± 2.12 Ba	113.03 ± 0.90 Ba	127.57 ± 1.87 Aa	133.18 ± 1.14 Aa	87.0	< 0.01
	*F*	448.0	270.0	312.0	244.0	367.0		
	*P*	< 0.01	< 0.01	< 0.01	< 0.01	< 0.01		
*T. granarium*	Ma	90.53 ± 1.32 Eb	99.41 ± 2.07 Db	108.40 ± 1.65 Cb	122.58 ± 1.10 Bb	137.35 ± 1.51 Ab	141.0	< 0.01
	Lamb	71.58 ± 0.59 Ed	84.58 ± 1.57 Dd	92.60 ± 0.92 Cd	103.72 ± 1.43 Bd	115.78 ± 1.96 Ad	151.0	< 0.01
	DE	81.60 ± 0.93 Ec	92.23 ± 1.58 Dc	99.65 ± 1.67 Cc	110.42 ± 1.88 Bc	126.78 ± 1.14 Ac	136.0	< 0.01
	Ma + DE	52.71 ± 1.33 Ee	67.15 ± 1.53 De	78.30 ± 1.09 Ce	92.80 ± 1.46 Be	103.40 ± 0.87 Ae	245.0	< 0.01
	DE + Lamb	38.41 ± 1.26 Ef	43.11 ± 0.82 Dg	54.13 ± 0.93 Cg	68.26 ± 0.75 Bg	79.40 ± 1.26 Ag	275.0	< 0.01
	Ma + Lamb	41.16 ± 2.23 Ef	52.50 ± 0.54 Df	65.78 ± 1.28 Cf	81.25 ± 1.17 Bf	87.53 ± 1.68 Af	167.0	< 0.01
	Control	112.37 ± 1.06 Ea	126.55 ± 1.69 Da	137.22 ± 0.63 Ca	155.37 ± 1.69 Ba	163.53 ± 1.45 Aa	231.0	< 0.01
	*F*	418.0	375.0	519.0	418.0	405.0		
	*P*	< 0.01	< 0.01	< 0.01	< 0.01	< 0.01		
*R. dominica*	Ma	53.21 ± 1.24 Db	57.50 ± 1.32 Db	63.88 ± 1.27 Cb	75.43 ± 1.31 Bb	81.26 ± 0.72 Ab	97.5	< 0.01
	Lamb	36.18 ± 1.04 Cd	38.23 ± 1.02 Cd	49.30 ± 0.97 Bc	67.31 ± 1.24 Ac	63.71 ± 1.24 Ad	164.0	< 0.01
	DE	41.53 ± 1.09 Dc	49.21 ± 1.34 Cc	61.50 ± 1.54 Bb	72.61 ± 0.70 Ab	74.11 ± 0.50 Ac	165.0	< 0.01
	Ma + DE	27.83 ± 1.73 De	35.21 ± 1.33 Cd	47.43 ± 1.68 Bc	56.45 ± 0.47 Ad	57.25 ± 0.60 Ae	103.0	< 0.01
	DE + Lamb	18.56 ± 0.95 Cf	21.58 ± 0.59 Cf	33.85 ± 0.60 Bd	35.40 ± 1.29 Bf	46.18 ± 1.20 Af	131.0	< 0.01
	Ma + Lamb	21.6 ± 10.63 Ef	28.20 ± 1.49 De	36.25 ± 1.07 Cd	43.30 ± 0.96 Be	54.23 ± 1.13 Ae	135.0	< 0.01
	Control	87.61 ± 1.05 Ea	96.30 ± 1.52 Da	107.70 ± 1.90 Ca	121.70 ± 1.58 Ba	128.83 ± 1.76 Aa	115.0	< 0.01
	*F*	426.0	387.0	337.0	609.0	626.0		
	*P*	< 0.01	< 0.01	< 0.01	< 0.01	< 0.01		

Within each row, means followed by the same lowercase letter are not significantly different; *df* = 4, 29, Tukey–Kramer (HSD) test at *P* = 0.05. For each species, within each column, means followed by the same uppercase letter are not significantly different; *df* = 6, 41, Tukey–Kramer (HSD) test at *P* = 0.05

## Discussion

In this study, the single and combined effects of the EPF *M. robertsii*, the pyrethroid lambda-cyhalothrin, and DE were investigated in pairs as wheat protectants against *T. castaneum*, *T. granarium*, and *R. dominica* in laboratory and persistence bioassays. The most efficient treatment regarding mortality in laboratory trials, persistence, and progeny production was DE + Lamb, followed by Mr + Lamb, and Mr + DE for all insect species tested. Single treatments resulted in significantly lower mortalities for all insect species tested when compared to joint treatments.

In the context of reducing pesticide application, the utilization of biopesticides, notably *M. robertsii*, has garnered increasing attention among the scientific community as a prospective solution for biological pest management in both agricultural fields and forest environments (Ansari and Butt 2012; Velavan et al. 2017; Reynoso-López et al. 2021; Iwanicki et al. 2021; Barzanti et al. 2023). As an EPF, *M. robertsii* has the advantage of greater residual persistence when compared to the majority of traditional insecticides (Moore et al. 2012). This was evident in the present study since, despite the lower mortality percentage caused by this EPF in laboratory trials across all insect species, its persistence exceeded lambda-cyhalothrin at 120 days of storage at the 7-day initial exposure. Pyrethroids have been instrumental in pest control over an extended period due to their effectiveness, knockdown effects, and limited environmental persistence. As a pyrethroid, lambda-cyhalothrin induces hyperactivity, incoordination, convulsions, and nerve depolarization blockage, ultimately resulting in the rapid demise of the insect (Becker et al. 2010; Palmquist et al. 2012). Consistent with prior research indicating the swift efficacy of lambda-cyhalothrin against diverse storage pests across different surfaces (Jankov et al. 2013; Sayed Abd Elkareem et al. 2015; Gharib et al. 2021), the current study's findings also underscore the remarkable effectiveness of this component in the mortality of the tested storage pests in laboratory trials. Jankov et al. (2013) who studied the efficacy of malathion, pirimiphos-methyl, and lambda-cyhalothrin on various surfaces against *Sitophilus oryzae* (L.) (Coleoptera: Curculionidae) demonstrated that lambda-cyhalothrin was the sole formulation that persisted for a duration of up to 180 days on concrete, distinguishing it from the other formulations. A toxicity test of lambda-cyhalothrin, malathion, carbosulfan, chlorpyrifos, fenitrothion, phosphamidon, cypermethrin, monocrotophos, and propoxur against *T. castaneum* highlighted lambda-cyhalothrin as the most toxic component against this notorious storage pest, underscoring its efficiency (Khalequzzaman and Nahar 2001). Apart from the effectiveness of DEs in several aspects, such as minimal development of insect resistance, strong adherence to grain, ease of removal from the grain, and low toxicity to mammals, DEs also exhibit a high level of persistence on the grain (Korunić et al. 2016; Losic and Korunic 2018; Audu and Ibrahim 2021). The present study has illustrated that while DE may not have displayed the highest mortality compared to lambda-cyhalothrin in the laboratory trials, this particular formulation outperformed lambda-cyhalothrin and *M. robertsii* after a 120-day storage period in all tested pest species. This held true for both the 7-day and 14-day initial exposure to the insecticides, highlighting the advantage of DEs in persisting within the grain.

The use of different combinations of insecticides can result in a range of outcomes in terms of pest mortality. This underscores the necessity for comprehensive exploration and optimization of these combinations. The combinations of alpha-cypermethrin with malathion, lambda-cyhalothrin, and clove oil against *R. dominica* have been shown to have various interactions concerning pest mortality, with alpha-cypermethrin + malathion exhibiting antagonistic interactions and the remaining combinations resulting in synergism (Hamza 2018). The combination of DEs with chemical pesticides, plant extracts, or entomopathogenic fungi has been demonstrated to significantly improve efficacy against storage pests when compared to individual applications of each insecticidal component (Michalaki et al. 2006; Losic and Korunic 2018; Rizwan et al. 2019; Hanif et al. 2022). Ashraf et al. (2017), who studied the efficiency of *M. robertsii* and DE in single and joint treatments against *Liposcelis paeta* Pearman (Psocodea: Liposcelididae)*, Cryptolestes ferrugineus* (Stephens) (Coleoptera: Laemophloeidae)*, R. dominica,* and *T. castaneum* on wheat, revealed that *M. robertsii* + DE demonstrated a synergistic effect in the mortality of the pests tested. In a study by Wakil et al. (2023a, b), the investigation into the individual and combined impacts of indoxacarb at 5 ppm, DE at 150 ppm, and the EPF *Beauveria bassiana* (Bals.-Criv.) (Hypocreales: Cordycipitaceae) at 1 × 10^7^ conidia/kg wheat revealed an improved effectiveness of the combined treatments. The authors revealed that indoxacarb induced the most pronounced mortalities among all the tested insect species in laboratory trials. Nonetheless, its persistence over 120 days of storage was comparatively lower in comparison to DE. Conversely, DE exhibited less immediate efficacy in causing mortality in the examined insect species but excelled in terms of persistence when contrasted with indoxacarb. The most effective combined treatment, as demonstrated, was the combination of the oxadiazine with DE. The findings of the current research align with the results of the aforementioned study, further emphasizing the efficacy of the EPF + DE combination to the immediate mortality of the tested insect species. In the context of interactions between each insecticide, the mode of action of each component significantly influences the resulting insect mortality. The high knockdown effect of lambda-cyhalothrin combined with the elevated grain persistence of DE rendered this combination the most successful in both laboratory and persistence trials of the current study. The elevated mortality observed in the tested pests holds profound significance since these species are known to exhibit tolerance to conventional insecticides (Rigaux et al. 2001; Athanassiou et al. 2011; Riaz et al. 2016). Furthermore, the sustained mortalities observed in persistence trials, even 120 days post-exposure to these combined treatments, at least for *R. dominica* underscore the potential viability of this combination for prolonged storage periods.

The efficacy of an insecticidal component as a means of pest management was effectively demonstrated through the considerable increase in mortality rates and a decrease in the number of offspring within the treated groups compared to the control group. This reduction in progeny was particularly pronounced among the treatments that resulted in higher mortality rates of the pests under examination. The progeny count was lowest in *R. dominica*, followed by *T. castaneum* and *T. granarium*, aligning with their respective mortality rates. While complete suppression of offspring was not achieved, it consistently remained significantly lower than in the control group for all insect species, whether they were subjected to single or combined treatments. This trend was uniformly observed in both the laboratory and persistence trials. The partial survival of parental adults may contribute to the emergence of progeny production in the insect species tested, given that achieving even 100% adult mortality does not consistently result in the complete eradication of offspring (Athanassiou et al. 2011; Kabir 2013).

The current study provides compelling evidence of the effectiveness of the paired combinations of lambda-cyhalothrin, DE, and *M. robertsii* as wheat protectants against three notorious storage pests known for their tolerance to conventional insecticides. These treatments demonstrated significant impacts on the mortality of the species under consideration both in laboratory and persistence trials. Notably, the combination of lambda-cyhalothrin with DE emerged as the most successful treatment, emphasizing its potential for pest management. These findings hold practical implications for reducing pesticide applications and advancing the use of biopesticides, contributing to the evolving field of pest management in storage. Nevertheless, additional research covering various commodities, temperature ranges, species, and doses is imperative to enhance and authenticate this approach as a comprehensive and dependable management tool for a wide range of storage pest scenarios.

## Data Availability

The datasets used and/or analyzed during the current study are available from the corresponding author on reasonable request.
